# Supplementary material for: Quantitative bioluminescence assay for measuring Bacillus cereus nonhemolytic enterotoxin complex
Source: PLoS One. 2020 Sep 30;15(9):e0238153. doi: 10.1371/journal.pone.0238153 (PMC7527251; doi:10.1371/journal.pone.0238153)
Supplement: Attachment — Submitted filename: 4Response to Reviewers.docx [file pone.0238153.s003.docx]

Dear Academic Editor Dr. Maria Gasset,

Thank you for considering our manuscript entitled: “Quantitative Bioluminescence Assay for Measuring *Bacillus cereus* Nonhemolytic Enterotoxin Complex” (ID: PONE-D-20-15755) for publication as a research article in PLOS ONE. We have addressed the questions posed by the Reviewers and would like to thank Reviewers for their constructive comments. We feel that the manuscript has greatly benefited as a result.

Reviewer #1 raised no objections to publication as is. Reviewer #2 raised the following points and we have addressed them as noted.

… the authors must answer important questions before approval for publication
1.- The scope of the article fits much better the scope of a Food technology journal rather than PLOS One, although it is a full decision from the Editor.

The authors recognize the broad scope of the journal PLOS ONE and the approach described may have broader analytic potential application. While the bioluminescence assay was demonstrated in the manuscript for measuring nonhemolytic enterotoxin activity, similar cell-based assays combined with low-cost optical sensing device can be adapted for the analysis of activity of other biomolecules and different type of toxins as an alternative to live animal testing. We added this important point to the discussion. We believe the broad potential application of the approach will be of interest to the journal’s readers.

2.- The information provided in the Introduction about the impact of *B. cereus* as a food spoilage in human heath are outdated, the authors are advised to provide more

We have substantially expanded the introduction, specifically including more discussion of the impact of *B. cereus* and its toxins on human health and the need for improved alternatives to live animal testing for assessment of the toxic potential of isolates and contaminated foodstuffs. We have expanded the reference set accordingly with recent literature on the subject. Food is routinely heat treated to kill contaminant microbial pathogens and to inactivate their toxins. The main drawback of PCR and ELISA assays is their inability to differentiate between active toxin from inactivated toxin. PCR assay can detect the presence of the genetic material of non-viable microbial pathogens encoding toxin long after heat treatment already killed them. ELISA assay detects the presence of one of the three toxin subunits which poses no threat by itself. The activity bioassay that we developed distinguishes between the biological active form of the toxin, which poses a threat, from the inactive form, which poses no threat.

2,.- No doubt, the aim of the authors is to remark the practical implementation of their technique, and the advance provided by their approach. In order to do that, the drawbacks for the other techniques, animal model, PCR and ELISA were described, but they did not compared the sensitivity of at least PCR and ELISA with their method. From my point of view, just the presence of Bacillus cereus regardless of the level of toxin production is by itself a serious threat in food contamination. Does the new procedure challenge these two other techniques in terms of sensitivity?

In terms of limit of detection, our bioluminescence assay can detect 7 ng/ml Nhe. This data compares with ELISA, PCR and lateral flow apparatus (LFA). ELISAs have a comparable detection limit to our assay with a range of 2-5 ng/ml and the LFA has a detection limit around 20 ng/ml [35]. It is difficult to determine a detection limit for PCR, but has been shown to produce a negative result in samples where the LFA will produce a positive result. This indicates that the detection limit of PCR is higher than that of 20 ng/ml. However, none of these methods demonstrate the presence of the biologically active form of Nhe and PCR and LFA produce only qualitative results. Further, commercially available tests only show the presence of one sub-unit of the Nhe complex. We have included further clarification of these points in the Introduction and Discussion.

3.- According to the protocol proposed transfection plus 72 h incubation with the toxin is required. Is this time frame affordable for groceries compared with ELISA or PCR?. Have the authors tried shorter time frames?.

A shorter time frame will reduce the sensitivity of the assay. For the application of measuring toxin activity in food, the speed may not be so relevant because at the time of the measurement the presence of the toxin can already be established by immunologic screening assays, the relevant question remaining is whether the detected toxin is active and presents a health risk or whether the toxin is inactive.

4.- The authors described the problems associated to the practical use of ELISA or PCR in terms of the facilities required, but for this method a cell culture facility is needed , and also the t instability of the reagent solution for luciferase, especially for ATP, its substrate, that required to be frozen till its immediate use is obviated. In order to provide a fair scenario for this technique, these drawbacks must to be included, and if possible, their feasible solutions commented in the text.

We have pointed out these additional requirement in the Discussion. However, it is worth stressing again that the method presented is more accurately described as an alternative to live animal testing for fully functional and active toxin and not as an alternative to PCR and ELISA which do not serve that purpose.

We thank the reviewers for their constructive comments.

Sincerely,

Reuven Rasooly, Ph. D.
